# Using minimum bootstrap support for splits to construct confidence regions for trees

**Published:** 2007-02-03

**Authors:** Edward Susko

**Affiliations:** Genome Atlantic, Department of Mathematics and Statistics, Dalhousie University, Halifax, Nova Scotia, Canada

**Keywords:** bootstrap support, splits, confidence regions, statistical tests, phylogeny

## Abstract

Many of the estimated topologies in phylogenetic studies are presented with the bootstrap support for each of the splits in the topology indicated. If phylogenetic estimation is unbiased, high bootstrap support for a split suggests that there is a good deal of certainty that the split actually is present in the tree and low bootstrap support suggests that one or more of the taxa on one side of the estimated split might in reality be located with taxa on the other side. In the latter case the follow-up questions about how many and which of the taxa could reasonably be incorrectly placed as well as where they might alternatively be placed are not addressed through the presented bootstrap support. We present here an algorithm that finds the set of all trees with minimum bootstrap support for their splits greater than some given value. The output is a ranked list of trees, ranked according to the minimum bootstrap supports for splits in the trees. The number of such trees and their topologies provides useful supplementary information in bootstrap analyses about the reasons for low bootstrap support for splits. We also present ways of quantifying low bootstrap support by considering the set of all topologies with minimum bootstrap greater than some quantity as providing a confidence region of topologies. Using a double bootstrap we are able to choose a cutoff so that the set of topologies with minimum bootstrap support for a split greater than that cutoff gives an approximate 95% confidence region. As with bootstrap support one advantage of the methods is that they are generally applicable to the wide variety of phylogenetic estimation methods.

## Introduction

Bootstrap support or bootstrap probability (BP), Felsenstein (1985), for splits in a tree are frequently presented in the estimated trees of phylogenetic studies. A great deal of certainty about the estimated topology is suggested when bootstrap support for all of the splits is large. In cases where some of the splits have low BP, however, a number of questions arise as to which and how many of the taxa were supported as being in another place in the tree and where they might alternatively be placed. The full bootstrap output provides information that can be used to answer these questions. We utilize this information by finding the set of all trees with minimum bootstrap support (minBP) for their splits greater than some given value. Our reason for focusing attention on minBP is that since a tree is defined by all of its splits, for it to be included in a confidence region, all of its splits should be well supported. Thus its minBP should be inline with what one expects from the true tree. In some cases few trees will be included in the set, suggesting that while there is some uncertainty that a subset of taxa was on one side of a split, there were only a few other places in the tree that it might have been placed. In other cases many trees will be included in this set, suggesting that for the splits with low BP, there was very little information about where some of the taxa might alternatively be placed.

**Figure 1:**
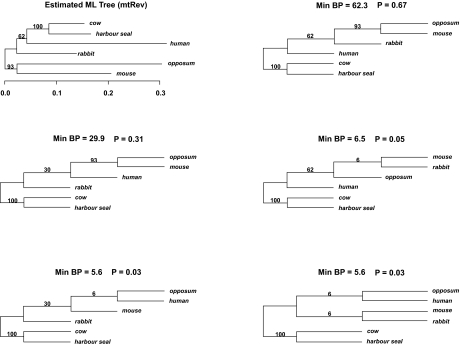
The estimated mammalian mitochondrial tree (first panel) with the top ranked trees in terms of minimum bootstrap support given across rows. Bootstrap support is indicated for each of the branches. Since the ranked trees are constructed from splits alone, branch lengths are arbitrary and taken as equal. Min BP is the minimum bootstrap support among splits in the tree. P gives the p-value for the null hypothesis that the tree is correct based on a double bootstrap procedure.

A brief example is given in Figure 1 for the mammalian mitochondrial data considered previously in Goldman, Anderson and Rodrigo (2000) and Shimodaira (2002). The estimated tree gives minimum BP to the split, corresponding to a relatively small branch length, that groups cow, harbour seal and human together. Out of the 1000 bootstrap samples, 623 supported this split. The top ranked trees in terms of minimum bootstrap support for a split (minBP) are given across rows; the branch lengths are arbitrary since these trees are calculated from splits alone. The first tree, not surprisingly, is the estimated tree; this need not be the case although with few taxa it is likely. The next tree has minBP 29.9% and corresponds to a nearest neighbour interchange of rabbit and human in the estimated tree. The third ranked tree corresponds to a nearest neighbour interchange of rabbit and mouse but has minBP only 6.5%. It is clear from the figures that only two topologies are well supported and that the reason for the 62.3% BP for the grouping of cow, harbour seal and human is because there is considerable uncertainty about whether the positions of human and rabbit should be switched. The splits with 93% and 62% BP in the estimated tree are small enough to create uncertainty about whether they are real. It is thus unclear, from only the estimated tree with BP for splits, whether a tree grouping human and mouse might be plausible. However, the first three trees in the Figure 1 give a 95% confidence region and none of these group together human and mouse, indicating that this hypothesis can be rejected. An idea implemented in Cooper and Penny (1997) provides useful supplementary to the estimated tree with BP. The 93% split becomes 93(99) where 99 is the summed BP for the split and nearest neighbour interchanges around that split. Since rabbit is the neighbour of opposum and mouse in the estimated tree, this presentation indicates that there is little support for a grouping of human and mouse. However, for more diverse splits ranging over several nearest neighbour interchanges, this device would no longer provide enough information to draw such inferences.

While methods to obtain a set of ranked trees based on minBP will be useful as supplementary information to BP for estimated splits, it is desirable to quantify the level of uncertainty associated with a set of trees through a confidence level. A (1 − α) × 100% confidence region for the true topology is a data-dependent, and hence random, set of topologies that contains the true topology with probability 1 − α. This differs from a Bayesian credible interval both in its construction and in that the true topology is treated as fixed but unknown, rather than random. Because of the duality between testing and confidence region construction, given a testing procedure, a (1 − α) × 100% confidence region can be constructed as the set of trees for which a test of the null hypothesis that the tree is correct gave a p-value ≥ α. Existing methods for testing whether topologies are correct include the the SH test (Shimodaira and Hasegawa 1999), the SOWH test (Swofford et al. 1996; Goldman, Anderson and Rodrigo, 2000), the GLS test (Susko 2003), the AU test (Shimodaira 2002) and the related complete-and-partial bootstrap test (Zharkikh and Li 1995). One immediate way in which a set of trees ranked by minBP can be converted to a confidence region is by using them as input to any one of these existing methods. In fact, since all of these tests require sets of candidate trees, the methods presented here supplement them by automating the construction of a set of candidate trees.

As an alternative to using a set of trees ranked by minBP as input to an existing testing procedure, we can treat minBP as a test statistic of the null hypothesis that a given tree is correct. This interpretation of minBP as a test statistic differs from the conventional interpretation as a p-value. In any case, as with any other test statistic, bootstrapping can be used to approximate its distribution. In effect this results in a double bootstrap since for each bootstrap sample, additional bootstrap samples are required to obtain the minBP value. A 95% confidence region of trees is given by the set of trees with minBP larger than the 5th percentile of the bootstrap distribution.

Considerable attention has been given to the question as to what constitutes large BP. Felsenstein (1985) and Felsenstein and Kishino (1993) consider 1-BP for a split as an approximate p-value for a test of the null hypothesis that the split is not present in the true tree. Considering simulation settings where the probability of estimating the correct tree is large (95%), Zharkikh and Li (1992) show that, with the criterion that a tree be accepted as correct when its BP is greater than 95%, the probability of failing to find any bifurcating tree satisfying this criterion it can be as low as 58%. Hillis and Bull (1993) indicate that BP is biased downwards if it is interpreted as an estimate of the probability that a true split will appear in the estimated tree; Newton (1996) gives reasons for this. Efron, Halloran and Holmes (1996) argue that the interpretation as an approximate p-value is correct up to an error of order $1/\sqrt{n}$, where *n* is the number of sites. Efron, Halloran and Holmes (1996) and Rodrigo (1993) use double bootstrapping to improve the accuracy of BP as an approximate p-value. This differs from the use of the double bootstrap here which is really a single bootstrap applied to a bootstrap test statistic. It also differs in that the null hypothesis of interest in each of these cases is that a split is not present while it is that a particular tree is correct for the methods presented here. The null hypothesis of a correct tree is considered in Shimodaira (2002) who uses multiple bootstrap samples of differing size to improve the accuracy of BP as an approximate p-value. In Shimodaira (2002), however, BP is the bootstrap support for a tree among a prespecified set of candidate trees. With large numbers of trees in this candidate set, which is likely to be the case with larger taxa sizes, BP for any given tree becomes very small and the procedure cannot be expected to work very well. What we attempt to exploit here is that BP for splits will continue to show variation in data settings where BP for trees has become too small and sparsely distributed.

The methods presented here are generally applicable to the wide variety of phylogenetic estimation methods. Required as input is a set of trees estimated in some way for the original data set, bootstrapped data sets and possibly double bootstrapped data sets when minBP-based confidence regions are desired. Software will be made available at http://www.mathstat.dal.ca/^~^tsusko that will produce files with the required bootstrapped data sets and, based upon the input estimated trees, will construct lists of a prespecified number of trees ranked according to minBP, calculate all trees with minBP at least as large as a cutoff and/or calculate the appropriate cutoff using the double bootstrap output.

## Methods

### Obtaining Sets of Trees From Sets of Splits

Buneman (1971) established systems of splits as an alternative and equivalent representation of a phylogenetic tree. A split corresponds to a branch or an edge on the tree and is a partition of the set of all taxa in the tree into two subsets or sides of taxa. For instance, in the first panel of Figure 1, the split corresponding to the branch with 62% BP has cow, harbour seal and human in one set *A* and rabbit, opposum and mouse in the other set, *A^c^*. A pair of splits *S*_1_ = *A*_1_ | *A*_1_*^c^*, *S*_2_ = *A*_2_ | *A*_2_*^c^*, are compatible if at least one of the pairwise intersections *A*_1_*A*_2_, *A*_1_*A*_2_*^c^*, *A*_1_*^c^* *A*_2_ and *A*_1_*^c^* *A*_2_*^c^* is the empty set. A phylogenetic tree satisfies that all of its splits are pairwise compatible and it is sufficient for a set of distinct splits to be pairwise compatible for it to define a tree. Since there are 2*m*-3 branches in an unrooted tree, where *m* is the number of taxa, 2*m-*3 distinct splits are required to define a tree.

Given a set of splits the algorithms presented here find all subsets of splits that define a tree. Splits corresponding to terminal branches can be ignored since they are present in all trees and compatible with all splits. It thus suffices to determine all subsets of *m* − 3 distinct pairwise compatible splits corresponding to internal branches (at least two elements on both sides of the split).

The discussion above was for binary trees. To restrict attention to such trees we treat a multifurcating tree as the set of binary trees with 0 edge lengths that gives rise to it. In terms of splits, a multifurcating tree is defined by *k* < *m* − 3 compatible splits and we represent it as the collection of *m* − 3 splits that are compatible and contain the *k* compatible splits.

Given a set of splits, *S_i_*, *i* = 1,…,*k* a simple algorithm that can be used is to find all sets of *r* compatible splits successively for *r* = 1,…,*m* − 3. The sets of compatible splits with *r* = *m* − 3 gives the set of trees. Given the sets of *r* compatible splits, *C*_1_,…,*C_s_*, the sets of *r* + 1 compatible splits are found by finding, for each of these sets, the set of splits compatible with it. The unions of the *C_j_* with the sets of splits compatible with them give the sets of *r* + 1 compatible splits. The difficulty with this approach is that the number of sets of *r* compatible splits can get very large for intermediate *r*.

An alternative algorithm updates a current set *C* = {*S_i_*_1_,…, *S_i_*_*_r_*_} (*i*_1_ < … < *i_r_*) of splits as follows

1. Obtain or update the set of splits, *P*, that are compatible with the splits in *C*.
2. If *r* < *m* − 4, add the first split in *P* to *C* and go to 1.
3. If *r* = *m* − 4, each split in *P* together with *C* gives a tree. Store the trees and update *C* by setting *S_i_*_*_r_*_ to the first split *S_i_*, *i* > *i_r_* compatible with the rest of the splits in *C*. Go to 1.

Much less storage is required for this algorithm but compatibility of splits requires checking more frequently. Some additional computational economies can be achieved. For instance, a *k* × *k* compatibility matrix, assigning a 1 or a 0 to the *ij* position according to whether *S_i_* and *S_j_* are compatible or not, can be constructed at the beginning so that further checks on compatibility require checking the *ij*th entry of the compatibility matrix alone. For the application of primary interest here, determining all trees with minBP at least as large as some threshold ζ(<0.5), any split with support larger than 1 − ζ, must be in the final tree and thus give a set of splits that can be placed in *C* initially and never removed.

Given a compatibility matrix, computation requires a sequence of binary comparisons to update the current set *C* and compatibility set *P*. The worst case time-complexity would arise when all choices of $\left(\begin{array}{c} k\\ m-3 \end{array}\right)$ splits from the *k* are compatible. In this case, each current set *C* of size *r* would have compatibility set *P* of size *k* − *r*. In addition, each of the possible $\left(\begin{array}{c} k\\ r \end{array}\right)$ compatible sets of size *r*, *r* < *m* − 4, would arise in the algorithm. For each of these, the update step 2 would check compatibility of the first split in *P* with the rest of the *k* − *r* − 1 splits in *P*. Thus the number of comparisons considered would be $\sum_{r=1}^{m-4}\left(\begin{array}{c} k\\ r \end{array}\right)\;(k-r-1)$ which indicates that the algorithm can be intensive when a large numbers of trees can be constructed from the initial set of splits.

### A Double Bootstrap as a Single Bootstrap

The approach we take here is to treat minBP not as a p-value but as a test statistic. A tree is included in a (1 − α) × 100% confidence region if its minBP is greater than the αth quantile of the distribution of minBP. Since the distribution of minBP is difficult to calculate analytically, a bootstrap must be used to approximate it.

Given an estimated tree, *B* nonparametric bootstrap samples are generated and, for each of these samples, the minBP value for the originally estimated tree is obtained giving *B* minBP values. The resulting αth quantile of the bootstrap distribution or α × *B*th sorted minBP value from the bootstrap samples is used as the cutoff for splits to include in obtaining the confidence region.

To outline why this use of the nonparametric bootstrapping gives a reasonable cutoff, it is useful to characterize the bootstrapping process as sampling of vectors of character states from the empirical distribution. Each bootstrapped data set is obtained by independently selecting vectors of character states (columns of a sequence alignment) from the probability distribution, *F̂*, that assigns probability 1/*n* to each of the observed vectors of character states (each of the positions in the alignment). Since the bootstrap generates data from *F̂*, the proportion of bootstrap samples with minBP larger than ζ should be approximately

(1) $$\begin{array}{l} P_{\hat{F}}(\text{min}\text{BP}>\zeta )\\ =P_{\hat{F}}(\text{BP}>\zeta ,\text{for all}\;\text{splits }s\;in\;\hat{T}) \end{array}$$

In principle, *B* can and should be chosen large enough that the proportion of minBP larger than ζ is equal to (1) up to a negligible error. In practice, particularly in the case of expensive phylogenetic estimation procedures, this is often not the case.

Under quite general conditions, *F̂*, the empirical distribution, converges to the actual distribution, *F*, of vectors of character states implied by the random substitution process and the true tree. We can usually think of the true tree in a variety of different ways as a function of *F, T*(*F*). For instance, *T*(*F*) is the tree that maximizes the expected log likelihood and *T*(*F*) is also the tree that minimizes the expected sum of squared pairwise distances. Which characterization is more appropriate depends on the estimation procedure under consideration. For instance, with likelihood estimation, thinking of *T*(*F*) as the maximizer of expected log likelihood is more appropriate, since the expected log likelihood for a single vector of character states from *F̂* is just the log likelihood, *T*(*F̂*) is the maximum likelihood estimate of the tree for the data. Since *F̂* ≈ *F* for large samples, the expectation is that

(2) $$\begin{array}{l} P_{\hat{F}}(\text{BP}>\zeta ,\text{for all splits }s\;\text{in }T(\hat{F}))\\ \approx P_{F}(\text{BP}>\zeta ,\text{for all}\;\text{splits }s\text{i}\text{n}\;T(F)) \end{array}$$

Since the left hand side of (2) is approximately the proportion of bootstrap samples with minBP larger than ζ, if the cutoff ζ is chosen as the αth quantile of the bootstrapped minBPs, we have

(3) $$\begin{array}{l} 1-\alpha =P_{\hat{F}}(\text{BP}>\zeta ,\text{for all splits }s\;\text{in}\;T(\hat{F}))\\ \approx P_{F}(\text{BP}>\zeta ,\text{for all splits }s\;\text{in }T(F))\\ =P_{F}(T(F)\;\text{in confidence region}) \end{array}$$

where the last equality follows from the fact that the true tree, *T(F)*, is in the confidence region if and only if all of its splits have BP greater than ζ. Thus the set of trees with minBP larger than the αth quantile of the bootstrap distribution gives an approximate (1 − α)×100% confidence region.

The use of bootstrapping actually requires a double bootstrap. For each bootstrap sample at the top level giving the minBP values for the estimated tree across bootstrap samples, additional bootstrap samples have to be taken to obtain the minBP values. This can quickly become costly. For the example applications here, we used 100 bootstrap samples in both levels of the bootstrapping process. Including the original data set, this requires 10101, estimations of trees.

## Results

The first two examples that we consider have been considered previously in the literature on confidence regions estimation and testing (Shimodaira and Hasegawa, 1999; Shimodaira, 2002; Goldman, Anderson and Rodrigo, 2000) and involve larger data sets and smaller numbers of taxa. The third, considers an archaebacterial elongation factor 1α(EF − 1α) data set and illustrates issues that arise with a larger number of taxa.

### Mammalian Mitochondrial Data

To determine a minBP cutoff for a 95% confidence region for the mammalian mitochondrial data considered earlier, 100 bootstrap samples were generated from the original data set. From each of these samples, another 100 bootstrap samples were generated to obtain the minBP value for the estimated tree. This resulted in 100 minBP values. The minBP cutoff was taken as the fifth percentile of the minBP values which turned out to be 6%; the minBP cutoff for a 90% confidence region was 15%. Based upon this, a 95% confidence region is given by the first 3 ranked trees with the third being a borderline inclusion

An alternative way of characterizing the support for a ranked set of minBP trees is through p-values. We can define a test of the null hypothesis that a given tree is the correct tree by rejecting that hypothesis at the α level if and only if the tree is not in a (1 − α)×100% confidence region. The p-value for this test is the smallest α level for which the null hypothesis can be rejected which, in the present case, is the smallest α for which the tree is not in a (1 − α)×100% confidence region. Since a tree is in the confidence region as long as its minBP is larger than the cutoff for that region, and the cutoffs are determined from the bootstrapped minBPs, the p-value is found by obtaining the largest bootstrapped minBP less than the minBP for the given tree. If this bootstrapped minBP is the *p*th quantile of the sample of bootstrapped minBPs then the p-value is *p*. For the mammalian mitochondrial data, the p-values are given in Figure 1.

Table 1 gives the p-values from Shimodaira (2002) for a number of topology tests. The 15 topologies are listed in Table 2 and are all of the topologies with cow and harbour seal split from the rest of the taxa. Here PP indicates approximate Bayesian posterior probabilities taken from Table 1 of Shimodaira (2001). BP is for the tree rather than a split. KH gives the p-values for the test of Kishino and Hasegawa (1989) and AU gives the p-values for the test of Shimodaira (2002). SH and WSH give p-values for the unweighted and weighted versions of the SH test (Shimodaira and Hasegawa, 1999; Shimodaira, 1993, Shimodaira, 1998; Buckley et al., 2001). The p-values for the GLS test are from Table 1 of Susko (2003). One can see that the minBP confidence region includes fewer topologies than most of the other methods, some of which, like the SH test, are known to be conservative. The p-values are most similar to the those from the BP test. Here BP for the tree is being used as a p-value. This is not very surprising since the two approaches are similar. However, with a larger number of potential trees than can arise with 6 taxa, it is reasonable to expect that bootstrap support for any given tree will be very small. In contrast, bootstrap support for splits will continue to show variation that can be used to distinguish between topologies.

## HIV Data

The HIV data set was considered previously in Goldman, Anderson and Rodrigo (2000) and Susko (2003). It consists of a set of six homologous sequences, each consisting of 2000 base pairs from the *gag* and *pol* genes for isolates of HIV-1 subtypes A, B, D and E: A1 (Q23), A2 (U455), B (BRU), D (NDK), E1 (90CF11697) and E2 (93TH057). For the GLS test and the minBP p-values, the F84 model as implemented in the PHYLIP package, Felsenstein, (1993), was used with gamma rate correction. The transition/transversion ratio was estimated as 4.70 and the α parameter for the gamma rate distribution was estimated as 0.23 in TREE-PUZZLE version 4.02 (Strimmer and von Haeseler 1996). The four topologies that had minBP greater than zero are listed in Table 3.

The particular null hypothesis of interest in Goldman, Anderson and Rodrigo (2000) and Susko (2003) was the hypothesis that the second topology listed in Table 3 was the true topology. For the SOWH and SH tests reported in Goldman, Anderson and Rodrigo (2000) the SOWH test gave a p-value of 0.002 and the SH, a conservative test, gave a p-value of 0.26. The GLS p-value reported in Susko (2003) was 0.005. The minBP for this topology was 9.5% giving a p-value of 0.01.

Interesting features of this analysis include a comparatively large minBP p-value, a large minBP cutoff of 31% for a 95% confidence region and the possibility that the F84 model is not flexible enough for the data. Given the results from other tests and the large drop in minBP from the first topology to the second, it is expected that increased double bootstrap sampling would reduce the p-value for all topologies except the first; with 100 bootstrap samples p-values are necessarily in the set {0.01,0.02,…,1.00}. However, a large number of bootstrapped minBP values would clearly be required. Without very significant computational resources, obtaining a fine resolution for minBP p-values is infeasible.

As discussed earlier, a double-bootstrap-free minBP cutoff is 5% and comes from the conventional interpretation of 1-BP as a p-value for the null hypothesis that a split is not present. In the present example this seems inappropriate. The results of the other tests indicate that there is very little support for the second and third topologies, which would have to be included with this cutoff, and the results of bootstrapping the minBP values indicate that values as small as 5% are quite unlikely.

An alternative approach to the nonparametric bootstrap is a parametric bootstrap where the bootstrapped minBP values are obtained from bootstrap data sets simulated from the fitted model with estimated parameters. A possible advantage of the nonparametric bootstrap is that since data is being generated from the empirical distribution it should be giving reasonable accurate approximations to the probabilities that minBP is larger than a value, even if the assumed substitution process is incorrect. If the tree estimation procedure is used with a misspecified model that is still “close enough” to the generating process to allow distinctions between competing topologies, reasonable results can be obtained. In the present case, an F84 model was used where Goldman, Anderson and Rodrigo (2000) used the more flexible GTR model. The similarity of results suggests that the model was “close enough”.

### Archaebacterial EF1-alpha Data

**Figure 2:**
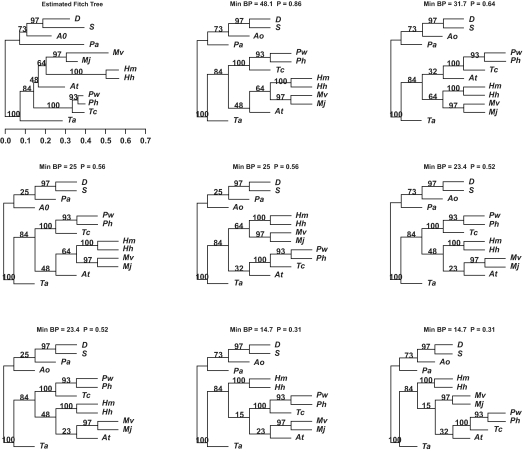
The estimated EF-1α tree (first panel) with the top ranked trees in terms of minimum bootstrap support given across rows. Bootstrap support is indicated for each of the branches. Since the ranked trees are constructed from splits alone, branch lengths are arbitrary and taken as equal. Min BP is the minimum bootstrap support among splits in the tree. P gives the p-value for the null hypothesis that the tree is correct based on a double bootstrap procedure.

With 13 taxa, the number of possible trees for the archaebacterial data set is 13,749,310,575, so that this data set serves to illustrate some of the difficulties in inference with larger numbers of taxa. The data set, which had 269 sites, was considered previously in Susko et al. (2003) and additional details are available there. Phylogenies were inferred by first estimating a maximum likelihood distance matrix using TREE-PUZZLE with an 8 category gamma distribution (DGE) model of rate variation and the PAM amino acid substitution matrix (Dayhoff and Eck, 1968; Dayhoff, Schwartz and Orcutt 1979). The Fitch-Margoliash method (implemented in FITCH, Felsenstein 1993) was used to infer trees from the distance matrices. There were 60 trees that had minBP larger than 5%, complicating summary of the information. The estimated tree and top 9 ranked trees, in terms of minBP, are given in Figure 2; longer names for the taxa are given in Table 4. Two other trees not indicated also had minBP 14.7.

The ranked trees, the fourth in particular, give an indication of reasons that the number of trees can be expected to grow quickly with larger numbers of taxa. The second ranked tree indicates that there is significant support for Af being closer to the Tc, Tw, Ph split. The third ranked tree indicates that there is support for the positions of Ao and Pa being switched. The fourth tree, combines both of these alternative splits, placing Af closer to the Tc, Tw, Ph split and switching the positions of Ao and Pa from what they were in the estimated tree. Generally, if a number of alternative splits are supported in separated regions of the tree, as is increasingly likely with larger numbers of taxa, any combinations of those splits will produce a tree that is well supported.

Based on a double bootstrap with 100 bootstrapped data sets at both levels, the cutoff minBP for a 95% confidence region was found to be 1% for this data set. Here, in contrast to the HIV data set, the cutoff was less than the 5% cutoff suggested by the BP test. Part of the reason for this has to do with the increased uncertainty about the tree for this data set. The small branch lengths and small BP suggest a generating tree that is closer to the “boundary” between trees in tree space, implying that BP is expected to be smaller for the generating tree. Even if the generating tree was a comparable distance from the boundary as for the HIV data set, because of the larger number of splits, the minimum BP over all splits can be expected to be smaller. The use of minBP as a test statistic adjusts for the multiple comparisons implied by considering a number of BPs instead of just one.

### Trees of Groups

**Figure 3:**
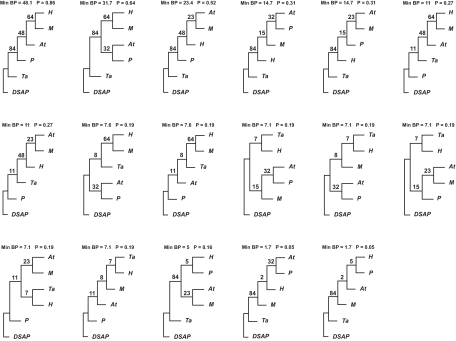
The EF-1α trees for groups Af, DSAP {D, S, Ao, Pa}, H {Hm, Hh}, M {Mj, Mv}, Ta and P {Tc, Ph, Pw}. All of the trees with minBP greater than or equal to 1 are indicated. The trees, ranked in terms of minBP, are given across rows.

The 264 trees with minBP greater than or equal to the 1% cutoff for a 95% confidence region is too large for easy presentation and some extraction of summary information from this set is required. A simple summary is provided by defining groups of taxa and presenting all trees in the confidence region that are compatible with those groups as trees of groups where taxa labels are replaced by group labels. An example is given in Figure 3 for the EF-1α data set with the groups Af, DSAP {D, S, Ao, Pa}, H {Hm, Hh}, M {Mj, Mv}, Ta and P {Tc, Ph, Pw}. The routine for obtaining this set of trees was obtained through the following. For each tree,

1. For each group, the split with the group on one side must be present in the tree for it to be compatible with the groups.
2. If the tree is compatible with the groups, for each group, a single representative is selected and the splits between representatives are determined.
3. The splits for the group representatives obtained in 2 give a tree for the groups with the names of the representatives replaced by the group names.

In the set of 264 trees for all of the taxa, 72 were incompatible with the groups indicated. Many more of them corresponded to the same tree with groups as taxa but with some variation of splits within groups.

Since the probability is 0.95 that the correct tree is contained in a 95% confidence region of trees, the probability that the correct tree of groups is contained in the set of distinct trees of groups corresponding to the trees in the region is 0.95 as well. Thus this set provides a confidence region for the trees of groups. This is true for confidence regions generally, not just those constructed using minBP. Note, however, that an assumption is being made about the existence of groups in the tree.

The above approach provides a way of extracting summary information when the confidence region of trees is large. However, the knowledge that groups exist can be used to create smaller confidence regions. The same arguments as were given in (1) and (2) apply with *T* replaced by splits of groups. In the original approach, the minimum bootstrap support is over both within and between groups splits, while in this approach the minimum is only over between group splits. Consequently the 5% cutoff coming from a double bootstrap can be expected to be larger and thus the confidence region of trees of groups will be smaller than the set extracted from the confidence set of trees.

In principle, estimation in this case should be constrained: trees should be estimated with the splits of groups present whether this is the case for the unconstrained estimated tree or not. As an approximation that avoids recomputing trees for every choice of groups, one can ignore bootstrap samples that give trees that are incompatible with the group splits. Since any samples where the groups were present in the tree give the same unconstrained estimate as the constrained estimate, and since these constitute a majority of the cases, the resulting minBP cutoff should be approximately the same as if the more appropriate constrained estimation had been used.

For the EF-1α data, the cutoff was found to be 1% as it was for confidence regions for the original trees. Thus the confidence region for groups of trees is given in Figure 3 as it was when these were extracted from the trees of taxa.

## Discussion

### Bootstrap Support for Trees

**Figure 4:**
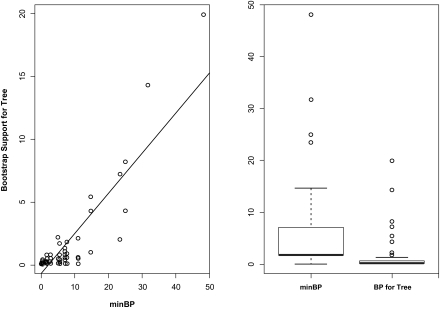
A scatter plot and boxplot of the minBP values and BP values for trees arising in 1000 bootstrap replicates for the EF1-α data.

The methods presented here are most closely related to a variety of methods that use bootstrap support for topologies to construct confidence regions, including those discussed in Sanderson (1989), Efron, Halloran and Holmes (1996), Rodrigo (1998), Zharkikh and Li (1995) and Shimodaira (2002). BP for a topology can alternatively be thought of as BP jointly for its splits. Since minBP is for a single split a natural concern is that some of the multivariate information in BP for topologies has been lost. This is a bit misleading since, for a tree to be included in the confidence region, all of its splits must have arisen with reasonable frequency (minBP must be above a threshold) and the splits must be compatible; these are properties the splits must jointly satisfy. This is indicated in Table 5 where for the mammalian mitochondrial and HIV data the BP and minBP for topologies with non-zero BP are indicated. In this case they are almost the same because there were so few topologies that were supported. As illustrated in Figure 4, for the EF-1α data the situation is a little different. The BP and minBP values correlate well but the BP values associated with the lower supported topologies show too little variation to make distinctions; there were 60 trees with less than 1% BP.

Other BP methods rank bootstrap support for trees in order of some distance from the “best” tree (cf Sanderson 1989), for instance, the majority rule consensus tree of the bootstrap trees. Difficulties with this approach include the choice of distance. In addition, Figure 1 of Rodrigo (1998) gives an interesting example where the symmetric distance between the estimated topology for a data set and an alternative is too small for the alternative to be rejected, even though the data clearly do not support the alternative. Nevertheless, this approach is well motivated as similar to the percentile method (cf Efron and Tibshirani 1993) for bootstrapping a mean. However, the resulting 95% confidence interval for the mean would include all values between the 2.5th and 97.5th percentiles of the bootstrap distribution; it would not be the discrete set of bootstrapped means that arose in bootstrap samples. By analogy, the BP methods that rank bootstrap support for trees in order of distance should include all trees that are within that distance not just those that occurred in bootstrap samples. This is particularly important when small numbers of bootstrap samples are taken (with 1000 bootstrap samples, at most 1000 trees can be in the region). The algorithms presented here provide a way of expanding the set of trees to be checked for small distance while at the same time restricting that set in a sensible way so that undo checking of trees that are unlikely to be included is not done.

Other ways of using BP include constructing the bootstrap profile, or set of all topologies that arose in bootstrap sampling (Rodrigo et al 1993; Rodrigo 1998). Calculation of the bootstrap profile has the advantage of not requiring determination of appropriate cutoff values for inclusion with the tradeoff that more trees are included than is necessary. It is useful as a conservative approach since if a topology is not included in the set, it would not be in the smaller set that only included topologies with BP above some threshold. This approach could be used with minBP as well which has the advantage of not requiring a double bootstrap for determination of minBP cutoffs. A similar approach considers the smallest set of topologies, ranked from highest to lowest BP, that give a cumulative total of 95% bootstrap support. Once again this is similar to the percentile method of bootstrapping that includes all parameter values, topologies in this case, that are in the highest density region of the bootstrap distribution of the parameters. It is interesting to note however that the cutoffs that result from this procedure will tend to be quite different from the 5% cutoff of the BP test discussed as first-order correct in Efron, Halloran and Holmes (1996) and Shimodaira (2002). For instance, for the EF-1α data, in 1000 bootstrap samples, there were 47 trees that appeared once and another 11 that appeared twice giving a cutoff of 0.2%.

In theory, if it were possible to calculate the limiting BP for each topology as the number of bootstrap samples increases without bound, this value should be used as BP. The fact that this cannot be done leads to difficulties in high-uncertainty problems as has been noted in Lutzoni (1997), Cunningham (1997) and Rodrigo (1998). If 1000 bootstrap samples are taken, at most 1000 trees can be included in the confidence region, even if the uncertainty present in the data is so great that 1,000,000 trees should be included. Using the cumulative total of 95% rule, if 800 topologies arose a single time in 1000 bootstrap samples, which should be included? Phrased in terms of limiting BP, when the appropriate but unknown confidence region contains 1,000,000 trees the BP for some of those trees must be at most 0.0001% and more than 1,000,000 bootstrap samples would be required to determine which trees these are. The use of minBP can prove useful here since the set of trees for 1000 samples can be larger than 1000 due to compatible splits. Every bootstrap sample that gives a tree gives all of its splits and so the minBP for a tree will always be bigger than the BP, whether with finite bootstrap samples or in the limit. In high-uncertainty problems, a topology that has non-zero limiting BP will have non-zero limiting minBP as well and the probability that its minBP will be positive in any given set of finite bootstrap samples will be larger than the corresponding probability for BP.

**Figure 5:**
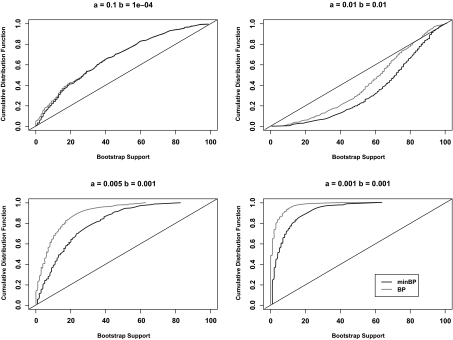
Plots of the cumulative distribution of bootstrap support for the two tree. Each curve gives the probability that bootstrap support is less than or equal to the corresponding quantity on the x-axis. Curves are given for both the bootstrap support of the topology and the minimum bootstrap support of the splits of that topology. The true generating tree was the same as the estimated tree for the mammalian mitochondrial data in Figure 1, but with branch lengths ((*seal*: 0.1, *cow*: 0.1): *a, human*: 0.1): *b, (rabbit*: 0.1, (*mouse*: 0.1, *opposum*: 0.1): *a*) The internal branch lengths *a* and *b* were allowed to vary. The cumulative distribution functions were estimated from 1000 nucleotide data sets simulated under a Jukes-Cantor process each with *B* = *100* bootstrap replicates.

High-uncertainty problems where large numbers of trees should be included in a confidence region can only arise with substantial numbers of taxa but difficulties with low BP can arise with small numbers of taxa as well if the true tree is poorly resolved. We illustrate this in Figure 5 where the cumulative distribution functions of minBP and BP for the true tree are plotted for a six taxon tree of the same shape as the estimated mammalian mitochondrial tree but with differing levels of resolution due to the smaller or larger middle branches *a* and *b*. Each cumulative distribution was approximated through simulation. The BP and minBP values for the true tree was calculated for 1000 simulations with Jukes-Cantor maximum likelihood estimation being applied to sequences of 1000 nucleotides simulated under a Jukes-Cantor substitution process; 100 bootstrap samples were considered in each simulation. The cumulative distribution function, with the y-value giving the probability that BP (minBP) is less than or equal to the corresponding x value, was estimated based on the observed proportions in the simulations. The appropriate bootstrap cutoff value for a 95% confidence region is the value of BP (minBP) such that the probability of being less than or equal to it is 0.05. If every topology with BP (minBP) greater than this value is included in the confidence region, the probability of type I error, not including the true topology in the confidence region, is 0.05.

One interesting set of observations comes by comparison with the interpretation of BP as a first-order correct p-value (Efron, Halloran and Holmes 1996; Shimodaira 2002). According to this interpretation, a cutoff of 5% is appropriate. The case that best corresponds with theory is when *a* = 0.1 and *b* = 0.0001. In the language of Efron and Tibshirani (1998) and Shimodaira (2002), in this case the true tree is close to the boundary of regions between topologies but, with only one branch being close to 0, not as near a boundary with a great deal of curvature as a tree that had more unresolved splits. Still, for this case the probability that the true tree will be included in a 95% confidence region if a 5% cutoff is used is estimated as 0.84 for BP and 0.87 for minBP. For the other cases of poorly resolved trees *a* = 0.005, *b* = 0.001 and *a* = 0.001, *b* = 0.001, the probabilities that the true tree will be included in a 95% confidence region using a 5% cutoff for BP (minBP) are 0.80 (0.63) and 0.21 (0.49). While this suggests that the 5% cutoff is generally too large, the well resolved case when *a* = 0.01 and *b* = 0.01 saw BP (minP) values greater than 5% 1000 (999) times in the 1000 simulations.

The other interesting observation comes from considering the cutoffs corresponding to a 95% confidence region: the 5th percentiles of the distributions. For the poorly resolved cases *a* = 0.01, *b* = 0.0001, *a* = 0.005, *b* = 0.001 and *a* = 0.001, *b* = 0.001 the cutoffs for BP (minBP) were 1 (2), 0 (1) and 0 (1). Note that 100 bootstrap samples were being considered in each simulation. Each of these simulations gives an unbiased estimate of the limiting BP (minBP) with infinite bootstrap samples. However, if the 5th percentile of the distribution of the target limiting BP (minBP) is between 0 and 1%, which is almost certainly the case here, 100 bootstrap samples are not sufficient to determine it. In short, in each of these poorly resolved cases, more bootstrap samples are required to have enough resolution to determine an appropriate BP cutoff for a 95% confidence region. However, because the cumulative distribution of minBP moves away from 0 faster than the distribution for BP, less bootstrap sampling effort is required to obtain the same resolution.

In practice, since the true topology is not known, the cumulative distribution functions of Figure 5 that in theory give the appropriate cutoffs cannot be calculated. However, since the empirical distribution of site patterns approximates the distribution based on the true but unknown topology, bootstrap sampling from it can be used to determine the appropriate cutoffs which is the essential idea behind the double bootstrap proposed here. It is valuable to note that these ideas are applicable to BP just as well as to minBP. In cases where the tree is not too poorly resolved there is some merit in considering the double bootstrap approach applied to BP, since there are concerns about a loss of multivariate information due to the restriction of attention to BP for splits. However, as the examples illustrate, in poorly resolved cases, more bootstrapping effort will generally be required to obtain an appropriate cutoff.

### Additional Comments

With or without a confidence region interpretation the bootstrap methods presented here provide useful supplementary information to bootstrap support values, indicating what kinds of alternative splits had reasonable levels of support. As a confidence region construction procedure, with smaller numbers of taxa the HIV and mammalian mitochondrial analyses gave results comparable to existing confidence region construction methods. Most such methods require input of sets of trees for construction of confidence regions. One alternative use of the sets of trees with minBP greater than a threshold is as input to some other confidence region construction method. It should be noted that in some cases this is not strictly justified. For instance, the theory motivating the SH and AU tests assume a fixed set of trees, not a data determined set. In contrast the SOWH and GLS methods include in their confidence sets all trees that have large likelihood or small generalized sum of squares respectively. While in principle these routines should include all trees meeting a certain criterion they can only recognize those trees that meet the criterion among the input trees. Using the set of trees with minBP larger than a fairly small threshold automates the search for trees that might be expected to be included.

An undesirable attribute of the minBP confidence region construction method is that it fails to use additional information such as likelihood or branch lengths. However, this is unavoidable for any method that can be used in conjunction with a wide array of different methods including parsimony, likelihood and distance methods. Still it seems reasonable to expect that confidence region methods that use additional information will give smaller confidence regions; results for the HIV and mammalian mitochondrial data sets suggest comparable inferences however. Another potential drawback is that very large sets of trees can result with larger numbers of taxa. In this case a list of a fixed number of trees with top-ranked minBP can still be useful in providing supplementary information to bootstrap support.

Inference with larger numbers of taxa generally requires careful consideration. A multiple comparisons issue arises in that with larger numbers of taxa there are larger numbers of splits and thus the probability of finding a false split with large bootstrap bootstrap support increases. The use of minBP adjusts for this multiple comparisons issue with the tradeoff that a larger region of trees result. Sanderson and Wojciechowski (2000) illustrate that corrected BP diverges more from first-order correct bootstrap support as taxon size increases suggesting the that large sample approximations require larger samples to be accurate with larger numbers of taxa. Finally, regardless of the confidence region procedure, with larger numbers of taxa, even perfectly accurate confidence regions for trees can be expected to be much larger and summary becomes a difficulty. This suggests increased taxon sampling can be problematic for inference, although Zwickl and Hillis (2002) show that taxon sampling can improve estimation. One way of avoiding some of the difficulties while gaining the benefits for estimation is through the extraction of trees of groups from confidence regions for trees of taxa as was illustrated in the EF-1α example.

An alternative approach to the nonparametric bootstrap is a parametric bootstrap where the bootstrapped minBP values are obtained from bootstrap data sets simulated from the fitted model with estimated parameters. A possible advantage of the nonparametric bootstrap is that it should give reasonably accurate approximations to the probabilities that minBP is larger than a value, if the approximation of independence across sites is not too rough but the substitution process is misspecified. The tradeoff is that if the modeling is correct, the parametric bootstrap distribution can be expected to give a less variable approximation to the true distribution of pattern probabilities. Assuming a large enough sample size, the empirical distribution and parametric bootstrap distribution should be comparable since they both consistently estimate the true distribution and so one expects comparable answers. From a practical standpoint, the main reason for not using parametric bootstrapping is that it requires clear model specification, which is not required for parsimony and some distance methods. In addition, some models, like the covarion models of Galtier (2001) Huelsenbeck (2002), have software for estimation but not for simulation.

The focus here has been on inference but the tree with maximum minBP might also be considered as an alternative estimate of topology. While in every example considered here, the tree with maximum minBP was the estimated tree this need not be the case. In fact the maximum minBP is interpretable as a majority rule consensus tree with data-dependent percentage equal to largest percentage for which a binary tree can be constructed (c.f. Margush and McMorris, 1981; and Bryant 2003 for a broad discussion of consensus methods).

## Figures and Tables

**Table 1:** The p-values for the hypothesis that the tree is correct for the 15 trees with cow and harbour seal split from the rest. Trees are ranked according to log likelihood values as in Table 3 of Shimodaira (2002) based upon fits using PAML (Yang, 1997) and are listed in Table 2. PP denotes approximate Bayes posterior probabilities, KH, AU, SH and WSH denote p-values from the KH, AU, SH and weighted SH tests. The minBP values for each tree is given as is the p-value based on bootstrapped minBP values from 100 bootstrap samples each using 100 bootstrap sample to obtain a minBP value.

Tree	PP	BP	KH	AU	SH	WSH	GLS	minBP	p-value
1	0.934	0.579	0.039	0.789	0.944	0.948	0.0410	62.3	0.67
2	0.065	0.312	0.361	0.516	0.799	0.791	0.0380	29.2	0.31
3	0.001	0.036	0.122	0.114	0.575	0.422	0.0353	1.3	0.02
4	0.000	0.013	0.044	0.075	0.178	0.210	0.0024	6.5	0.05
5	0.000	0.035	0.066	0.128	0.149	0.299	0.0013	5.6	0.03
6	0.000	0.005	0.049	0.029	0.114	0.105	0.0050	5.6	0.03
7	0.000	0.017	0.051	0.101	0.112	0.252	0.0013	1.4	0.02
8	0.000	0.001	0.032	0.009	0.073	0.050	0.0050	1.0	0.01
9	0.000	0.000	0.003	0.000	0.032	0.015	0.0024	0.0	0.00
10	0.000	0.003	0.019	0.028	0.034	0.124	0.0013	1.0	0.01
11	0.000	0.000	0.010	0.003	0.018	0.069	0.0013	0.0	0.00
12	0.000	0.000	0.003	0.001	0.006	0.033	0.0013	1.3	0.02
13	0.000	0.000	0.003	0.001	0.006	0.034	0.0013	0.0	0.00
14	0.000	0.000	0.001	0.005	0.003	0.013	0.0013	1.0	0.01
15	0.000	0.000	0.001	0.002	0.002	0.009	0.0013	1.0	0.01

**Table 2:** The topologies for the 15 trees in Table 1.

Tree	Topology
1	((human,(seal,cow)),rabbit),mouse,opossum
2	(human,((seal,cow),rabbit)),mouse,opossum
3	(human,rabbit),(seal,cow),(mouse,opossum)
4	(human,(seal,cow)),(rabbit,mouse),opossum
5	human,((seal,cow),(rabbit,mouse)),opossum
6	human,(((seal,cow),rabbit),mouse),opossum
7	(human,(rabbit,mouse)),(seal,cow),opossum
8	(human,mouse),((seal,cow),rabbit),opossum
9	((human,(seal,cow)),mouse),rabbit,opossum
10	((human,mouse),rabbit),(seal,cow),opossum
11	((human,rabbit),mouse),(seal,cow),opossum
12	((human,mouse),(seal,cow)),rabbit,opossum
13	human,(((seal,cow),mouse),rabbit),opossum
14	(human,rabbit),((seal,cow),mouse),opossum
15	(human,((seal,cow),mouse)),rabbit,opossum

**Table 3:** The topologies with minBP larger than zero for the HIV data set.

Topology	minBP	p-value
A1,(A2,(E1,E2)),(D,B)	83.7	0.47
A2,(A1,(E1,E2)),(D,B)	9.5	0.01
A1,A2,((D,B),(E1,E2))	6.8	0.01
E2,(E1,(A1,A2)),(D,B)	2.0	0.01

**Table 4:** Full names for the 13 taxa in the archaebacterial EF-1 data set.

S	*Sulfolobus solfataricus*
D	*Desulfurococcus mobilis*
Ao	*Aeropyrum pernix*
Pa	*Pyrobaculum aerophilum*
Tc	*Thermococcus celer*
Ph	*Pyrococcus horikohii*
Pw	*Pyrococcus woesei*
Af	*Archaeoglobus fulgidus*
Mj	*Methanococcus jannaschii*
Mv	*Methanococcus vannielii*
Hh	*Halobacterium halobium*
Hm	*Haloarcula marismotui*
Ta	*Thermoplasma acidophilum*

**Table 5:** Bootstrap support and minimum bootstrap support for trees arising in 1000 bootstrap replications for the HIV and mammalian mitochondrial data.

HIV		Mammal	
BP	minBP	BP	minBP
83.7	83.7	59.2	59.9
9.5	9.5	33.7	33.8
6.6	6.8	5.6	5.6
0.2	0.2	0.7	0.8
		0.6	1.3
		0.1	0.8
		0.1	0.1

## Abbreviations

BP: bootstrap support
EF-1α: elongation factor 1α
minBP: minimum bootstrap support for a split
